# FKBP25 Regulates Meiotic Apparatus During Mouse Oocyte Maturation

**DOI:** 10.3389/fcell.2021.625805

**Published:** 2021-01-21

**Authors:** Danni Wang, Hongzheng Sun, Jiaqi Zhang, Zhenyue Huang, Congyang Li, Longsen Han, Yongan Xin, Shoubin Tang, Juan Ge, Qiang Wang

**Affiliations:** ^1^State Key Laboratory of Reproductive Medicine, Suzhou Municipal Hospital, Nanjing Medical University, Nanjing, China; ^2^Center for Global Health, School of Public Health, Nanjing Medical University, Nanjing, China

**Keywords:** oocyte, meiosis, FKBP25, maternal aging, reproduction

## Abstract

FK506 binding proteins 25 (FKBP25) has been shown to function in ribosome biogenesis, chromatin organization, and microtubule stability in mitosis. However, the role of FKBP25 in oocyte maturation has not been investigated. Here, we report that oocytes with FKBP25 depletion display abnormal spindle assembly and chromosomes alignment, with defective kinetochore-microtubule attachment. Consistent with this finding, aneuploidy incidence is also elevated in oocytes depleted of FKBP25. Importantly, FKBP25 protein level in old oocytes is significantly reduced, and ectopic expression of FKBP25 could partly rescue the aging-associated meiotic defects. In addition, by employing site-specific mutagenesis, we identify that serine 163 is a major, if not unique, phosphorylation site modulating the action of FKBP25 on meiotic maturation. In summary, our data indicate that FKBP25 is a pivotal factor for determining oocyte quality, and may mediate the effects of maternal aging on female reproduction.

## Introduction

Oocyte development is crucial in establishing female fertility. All of the oocytes protract arrest in prophase of the first division in the ovary, which can last decades in human (Keefe et al., 2015; Verlhac and Terret, 2016). Upon luteinizing hormone (LH) stimulation during puberty, immature oocytes resume meiosis, and undergo nuclear maturation characterized by germinal vesicle breakdown (GVBD). Along with progression of the chromatin condensation and microtubule (MT) organization, the oocytes gradually proceed to maturation, extruding the first polar body (Pb1). Oocytes then are arrested at metaphase of the second meiotic division, waiting for fertilization (Gosden and Lee, 2010). Meiotic maturation includes two important events, spindle assembly and chromosome movement, where any error in this process may lead to the generation of aberrant oocytes, such as aneuploid eggs (Chiang et al., 2012). In human beings, fertilization of aneuploid eggs is a major cause of female sterility, which increases with advancing maternal age (Hassold and Hunt, 2001). It has been demonstrated that maternal age-related decline in oocyte quality is associated with meiotic defects (Nagaoka et al., 2012; Holubcova et al., 2015; Duncan et al., 2017). Aged oocytes exhibit significantly changed spindle organization or MT dynamics, leading to kinetochore–microtubule attachment defects or chromosome segregation errors, which is the main reason for the meiotic failure (Li et al., 2017; He et al., 2019). Therefore, poor quality oocytes are an insurmountable problem for aged women to obtain optimal reproductive outcome (Ciancimino et al., 2014). Even though this is a clinically significant issue, strategies to improve oocyte quality with age have been scarce.

FK506 binding proteins (FKBPs), a large family of proteins, possess peptidyl prolyl *cis/trans* isomerase (PPIase) domains (Wochnik et al., 2005; Nelson et al., 2006; Liu et al., 2014). Fifteen FKBPs are discovered in the human proteome and family members (Dilworth et al., 2012; Galat and Thai, 2014; Galat et al., 2014). FKBPs are involved in the regulation of MTs organization and related to protein folding pathologies (Chambraud et al., 2007; Hausch, 2015). FKBP25, shuttling between the cytoplasm and the nucleus, closely related to histone modification enzymes, is a nucleic acid binding immunophilin (Gudavicius et al., 2013, 2014; Prakash et al., 2016). Structurally, FKBP25 contains a unique *N*-terminal Basic Tilted Helical Bundle domain (BTHB), tethered by a 54-amino acid flexible linker region to a *C*-terminal conserved FKBP domain (Helander et al., 2014; Dilworth et al., 2017). Numerous studies have described the association between FKBP25 expression and the regulation of ribosome biogenesis (Jin and Burakoff, 1993), chromatin, and MTs (Yang et al., 2001; Yao et al., 2011). Notably, FKBP25 has been demonstrated as a microtubule-associated protein (MAP), which is critical for maintaining the MT stability during mitotic progression (Dilworth et al., 2018).

Recent studies have shown that FKBP25 is phosphorylated by Protein Kinase C the key DNA binding sites during mitosis (De et al., 2014; Dilworth et al., 2018). These interactions are controlled by carefully timed phosphorylation events to ensure proper cell cycle progression and faithful chromosome segregation. To date, however, the role of FKBP25 in meiosis is not known. In this study, we discovered that depletion of FKBP25 protein has an adverse effect on the meiosis of mouse oocytes, especially disrupting the assembly of meiotic apparatus. Meanwhile, We found that FKBP25 expression was decreased in aged oocytes, which is associated with the spindle defects and chromosome misalignment.

## Results

### Localization of FKBP25 During Oocyte Maturation

**FIGURE 1:**
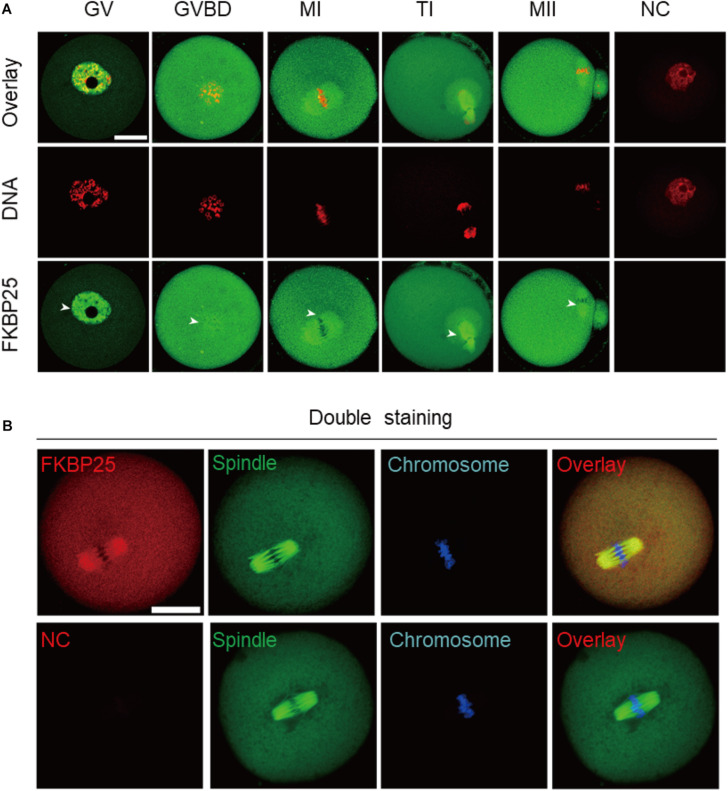
Cellular localization of FKBP25 in mouse oocyte. **(A)** Freshly collected oocytes of each stages were stained with FKBP25 antibody. Arrowheads point to the accumulation of FKBP25 signal. NC, negative control. **(B)** Metaphase oocytes were double labeled with FKBP25 antibody (red) and α-tubulin antibody (green), DNA was counterstained with Hoechst 33342 (blue). NC, negative control. Scale bar: 30 μm.

We firstly examined the localization of FKBP25 at different meiotic stages. As shown in Figure 1A, FKBP25 is predominantly distributed in the nucleus at GV stage. However, with the meiotic resumption, FKBP25 resides in the cytoplasm and significantly accumulated on the spindle region from pre-metaphase I (Pre-MI) to metaphase II stage (arrowheads). Furthermore, we confirmed that FKBP25 was colocalized with the spindle in oocytes by performing the double staining (Figure 1B). The dynamic distribution pattern implies that FKBP25 may play an important role in regulating oocyte meiotic maturation.

### FKBP25 Knockdown Adversely Affects Mouse Oocyte Maturation

**FIGURE 2:**
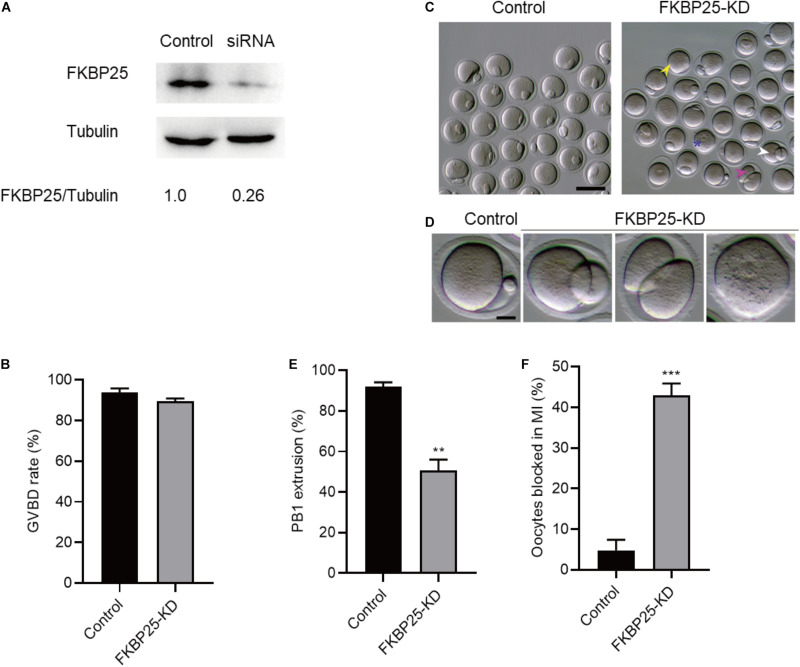
Effects of FKBP25 knockdown on oocyte maturation. **(A)** Western blot shows the knockdown efficiency of FKBP25 siRNA, 100 GV oocytes per sample. **(B)** Analysis the GVBD rate of control (*n* = 163 from 5 mice) and FKBP25-KD (*n* = 156 from 8 mice) oocytes. **(C)** Phase-contrast images of control and FKBP25 knockdown (FKBP25-KD) oocytes. Yellow arrowheads show the oocytes that fail to extrude polar bodies, pink arrowheads denote the oocytes with apparent symmetric division, blue asterisks point the oocytes arrested at GV stage, and white arrowheads indicate the oocytes with large polar bodies. Scale bar: 100 μm. **(D)** Magnified images for the abnormal oocytes shown in **(C)**. **(E)** Analysis the Pb1 extrusion rate of control (*n* = 163 from 5 mice) and FKBP25-KD (*n* = 156 from 8 mice) oocytes. **(F)** The percentage of oocytes arrested at metaphase I stage after FKBP25-KD injection (*n* = 124 for control from 4 mice; *n* = 120 for FKBP25-KD from 6 mice). Data are expressed as mean ± SEM. **p < 0.01, ***p < 0.001 vs controls.

To investigate the function of FKBP25 in meiosis, GV stage oocytes were microinjected with FKBP25 siRNAs. After injection, the GV oocytes were cultured in M16 medium supplemented with milrinone for 20 h to facilitate endogenous *FKBP25* mRNA degradation. The amount of FKBP25 protein level was dramatically decreased following knockdown (KD; Figure 2A). The results showed that FKBP25 knockdown had no effect on GVBD, shown by similar GVBD rate (Figure 2B). In contrast, FKBP25-KD oocytes exhibited a lower Pb1 extraction rate compared with controls after 14 h of mature culture (Figures 2C–E, asterisks). Oocytes with abnormal division, particularly large polar body extrusion, were frequently observed when FKBP25 was knocked down (Figures 2C,D, arrowheads). In addition, FKBP25-KD oocytes were significantly arrested at MI stage as compared with controls identified by immunofluorescence staining (Figure 2F). These observations strongly suggest that FKBP25 is required for meiotic divisions.

### Disorganization of Meiotic Apparatus in FKBP25-Depleted Oocytes

**FIGURE 3:**
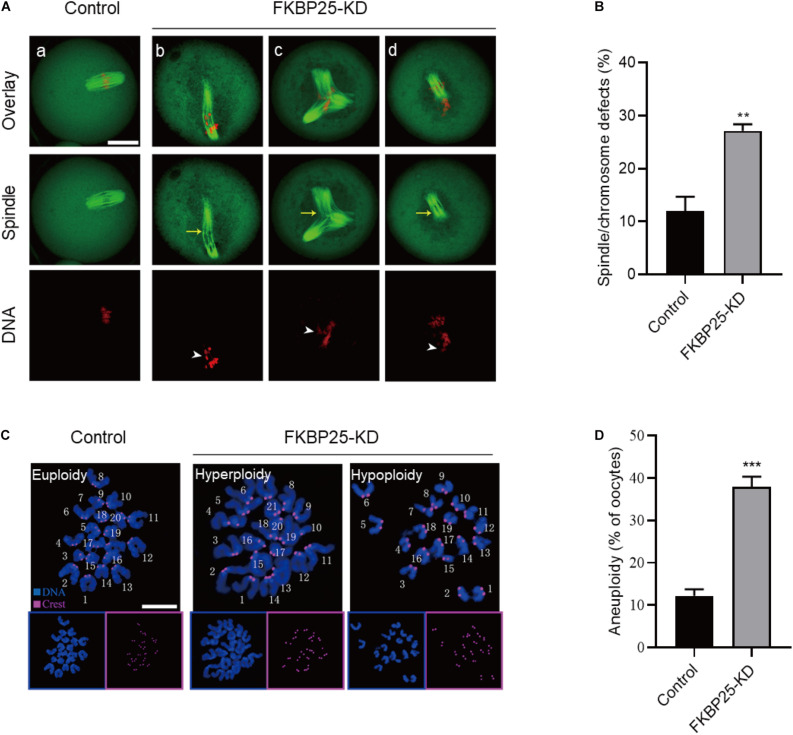
Morphological analysis of spindle and chromosome in FKBP25 knockdown oocytes. **(A)** Control and FKBP25 knockdown (FKBP25-KD) oocytes were stained with both α-tubulin antibody and PI. Disorganized spindles (yellow arrows) and misaligned chromosomes (white arrowheads) were frequently observed in FKBP25 knockdown (FKBP25-KD) oocytes. Scale bar: 30 μm. **(B)** Quantification of control (*n* = 124 from 4 mice) and FKBP25-KD (*n* = 120 from 6 mice) oocytes with spindle defects or chromosome misalignment. **(C)** Chromosome spread of control and FKBP25 knockdown (FKBP25-KD) oocytes. Chromosomes were labeled by Hoechst 33342 (blue) and kinetochores were stained with CREST (purple). Scale bar: 10 μm. **(D)** Quantification of aneuploidy in control (*n* = 51) and FKBP25 knockdown (*n* = 45) oocytes. Data are expressed as mean percentage ± SEM. **p < 0.01, ***p < 0.001 vs controls.

The specific localization of FKBP25 in meiotic process prompted us to investigate whether FKBP25 knockdown affects the meiotic apparatus in oocytes. To gain insight into this issue, we stained oocytes with anti-tubulin antibody and propidium iodide (PI) to visualize spindle and chromosomes, respectively. Using confocal microscope, we found a high defect rate in spindle assembly and chromosome organization in FKBP25-KD oocytes, showing the elongated/multipolar spindles (yellow arrows) and scattered chromosomes (white arrowheads; Figures 3A,B). By contrast, MII oocytes in control groups generally showed a typical barrel-shape spindle and well-aligned chromosomes. Moreover, the karyotype of MII oocytes was analyzed by chromosome spreading and kinetochore labeling. As shown in Figures 3C,D, we found that FKBP25 depletion resulted in about 3-fold increase in incidence of aneuploidy compared to controls. These results suggest that FKBP25 depletion disrupts spindle/chromosome organization in meiotic process, elevating the incidence of aneuploidy.

### FKBP25 Knockdown Impairs Kinetochore-Microtubule Attachments

**FIGURE 4:**
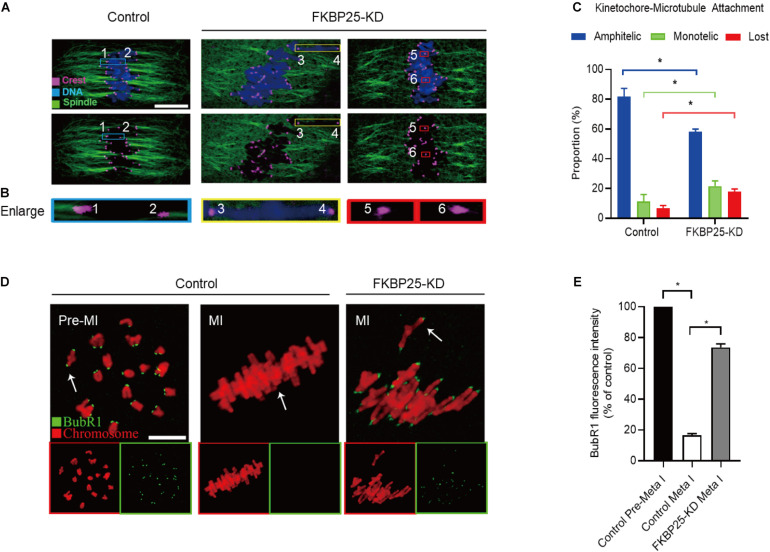
Depletion of FKBP25 in oocytes impairs kinetochore-microtubule attachments. **(A)** Metaphase oocytes in control and FKBP25-KD groups were double labeled with CREST and α-tubulin antibody. Representative confocal images are displayed. **(B)** Magnified images for the kinetochore-microtubule attachments in the oocytes shown in **(A)**. **(C)** Quantitative analysis of K-MT attachments in oocytes (*n* = 52 for control; *n* = 56 for FKBP25-KD). **(D)** Control and FKBP25-KD oocytes were stained with anti-BubR1 antibody (green) and counterstained with PI to examine chromosomes (red). Scale bar: 10 μm. **(E)** BubR1 fluorescence intensity in control (*n* = 45) and FKBP25-KD (*n* = 50) oocytes were quantified. Data are expressed as mean percentage ± SEM. *p < 0.05 vs controls.

Kinetochore, an important target connecting spindles and chromosomes, within a right location ensures that chromosomes are aligned at the equator and segregation precisely. Stable kinetochore-microtubule (K-MT) attachment is qualified to the spindle assembly checkpoint (SAC; Cheeseman, 2014; Tauchman et al., 2015). Given the disorganization of chromosome/spindle in FKBP25-KD oocytes, we hypothesized that FKBP25 knockdown may affect K-MT attachments. For this purpose, the kinetochores, MTs and chromosomes of MI oocytes were visualized by staining with anti-CREST antibody, anti-tubulin antibody and Hoechst 33342, respectively (Figures 4A–C). In most control oocytes, kinetochores were properly attached to MTs (amphitelic attachment). However, a prominently increased incidence of two forms of mismatch (lost attachment and monotelic attachment) was detected in FKBP25-KD oocytes relative to control oocytes (Figure 4C).

Spindle assembly checkpoint, a ubiquitous trigger that ensures the precise separation of chromosomes, monitors the right connections between kinetochores and MTs. The SAC signal is strongly expressed when the kinetochores is not yet integrated with the MT. When the kinetochores fused with the tubulin, the signal is quenched (Sun and Kim, 2012; Polanski, 2013; Sacristan and Kops, 2015). BubR1 (budding uninhibited by benzimidazole-related 1), an important integral element of the SAC, is often used to evaluate the status of SAC (Sudakin et al., 2001; Lara-Gonzalez et al., 2012; Overlack et al., 2015; Touati and Wassmann, 2016). In normal oocytes, BubR1 was detected during pre-MI, and then disappeared at MI stage. However, in FKBP25-KD oocytes, BubR1 signals were still present at MI stage (Figures 4D,E). Together, the defective K-MT attachments may be the main reason why chromosomes cannot align properly in FKBP25-KD oocytes.

### FKBP25 Overexpression Alleviates the Meiotic Defects in Old Oocytes

**FIGURE 5:**
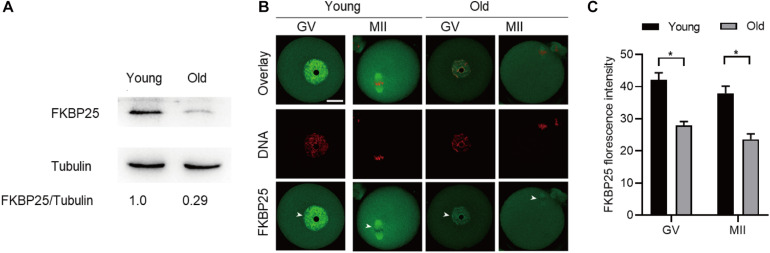
Expression of FKBP25 protein in oocytes from old mice. **(A)** Representative immunoblotting detecting the FKBP25 expression in oocytes from young and old mice (young, 100 oocytes from 3mice; old, 100 oocytes from 5 mice). **(B)** Confocal sections of young and old oocytes were immunolabeled with FKBP25 antibody (green) and counterstained with propidium iodide (PI) to visualize DNA (red). **(C)** Quantification analysis of FKBP25 fluorescence (27 young oocytes and 22 old oocytes for each experiment). Scale bar: 30 μm. *p < 0.05 vs controls.

**FIGURE 6:**
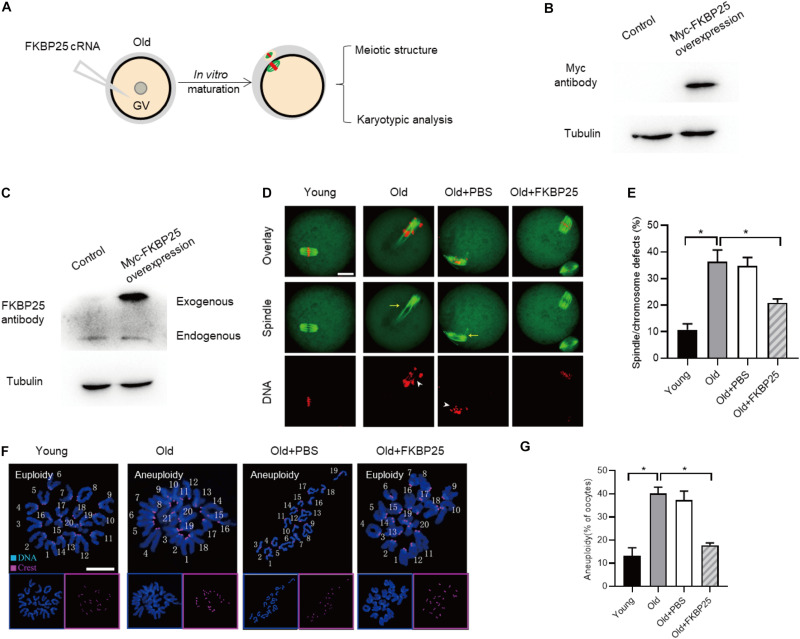
FKBP25 overexpression alleviates maternal age-induced meiotic defects. **(A)** Schematic illustration of the FKBP25 overexpression experiments. **(B,C)** Immunoblotting analysis detecting the exogenous FKBP25 expression. **(D)** Oocytes were stained with α-tubulin and PI to detect spindle and chromosome morphology, respectively. Arrows indicate the disorganized spindles and arrowheads indicate the misaligned chromosomes. Scale bar: 30 μm. **(E)** Quantification of the spindle/chromosome defects in each group (young, *n* = 113 from 5 mice; old, *n* = 102 from 7 mice; old+PBS, *n* = 105 from 10 mice; old+FKBP25, *n* = 124 from 10 mice). **(F)** Chromosome spread of oocytes in each group. Chromosomes and kinetochores were stained with Hoechst 33342 (blue) and CREST (purple), respectively. Scale bar: 10 μm. **(G)** Quantification of aneuploidy in young (*n* = 39), old (*n* = 30), old+PBS (*n* = 32), and old+FKBP25 (*n* = 28) oocytes. Data are expressed as mean percentage ± SEM. *p < 0.05 vs controls.

It has been well elucidated that female fertility decreases with maternal age on account of the maturation defects in oocytes (Holubcova et al., 2015; Duncan et al., 2017; Miao et al., 2020). Given that the FKBP25-KD oocytes exhibited the similar phenotypes as those in old oocytes, we decided to detect FKBP25 expression in oocytes from young and old mice. As shown in Figure 5A, remarkable reduction of FKBP25 protein was detected in oocytes from old mice compared with young oocytes at GV stage. We also assessed the FKBP25 distribution and accumulation in old oocytes via immunostaining. There was no significant alteration in the distribution pattern of FKBP25 between young and old oocytes (Figure 5B). However, the average florescence intensity of FKBP25 protein was dramatically decreased in both GV and MII oocytes from old mice (Figure 5C). In order to evaluate the relationship between FKBP25 protein level and the phenotypes of old oocytes, we performed the rescue experiments through microinjection of cRNA encoding FKBP25 into fully grown old oocytes (Figure 6A). Exogenous FKBP25 protein was efficiently expressed in oocytes (Figures 6B,C). It is worth noting that ectopic expression of FKBP25 ameliorated the maturation defects in old oocytes (Figures 6D,E). Furthermore, as shown in Figures 6F,G, overexpression of FKBP25 in old oocytes significantly reduced the incidence of aneuploidy. These results indicate that depletion of FKBP25 is one of key factors inducing meiotic defects in oocytes from aged mice.

### FKBP25 Phosphorylation Is Important for Modulating Meiosis in Old Oocytes

**FIGURE 7:**
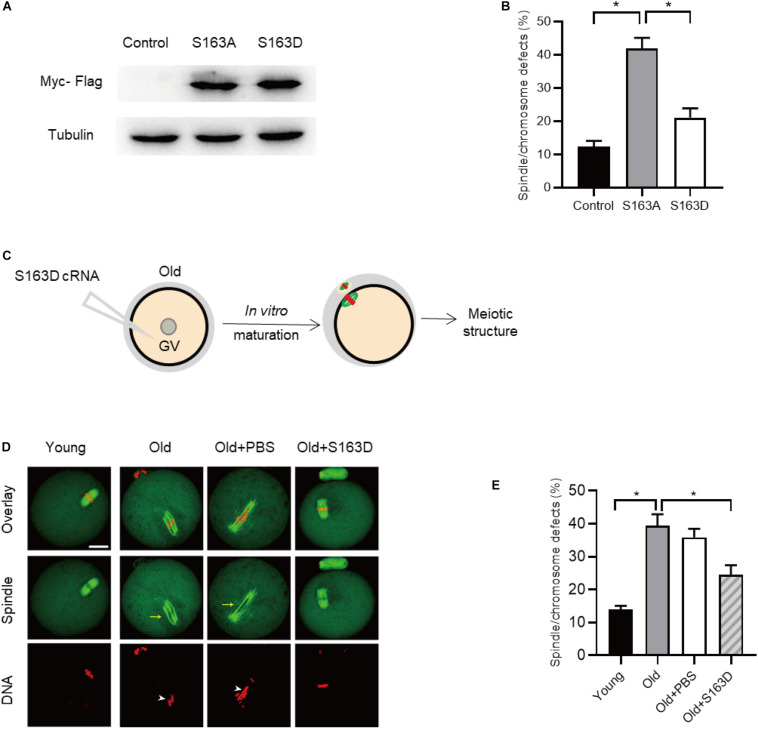
FKBP25-S163D mutant partly rescues meiotic defects in old oocytes. **(A)** Immunoblotting analysis detecting the expression of two FKBP25 mutants. **(B)** The histogram shows the effects of FKBP phosphorylation mutants on meiotic apparatus in oocytes (*n* = 95 for each group). **(C)** Schematic diagram showing the experimental design process. **(D)** Oocytes in each group were labeled with α-tubulin antibody (green) for spindle and PI (red) for chromosome. Arrowheads point to the chromosome misalignment and arrows point to the spindle disorganization. Scale bar: 30 μm. **(E)** Quantification of oocytes with spindle/chromosome defects in each group (young, *n* = 121 from 5 mice; old, *n* = 108 from 7 mice; old+PBS, *n* = 102 from 10 mice; old+FKBP25, *n* = 132 from 10 mice). Data are expressed as mean percentage ± SEM. *p < 0.05 vs controls.

During oocytes maturation, there is a dramatically change in protein phosphorylation and dephosphorylation. A wealth of these phosphorylation and dephosphorylation events are key to regulate meiotic process, chromosome dynamics, and spindle assembly (McGinnis and Albertini, 2010; Schindler, 2011). To test whether FKBP25 phosphorylation affects meiotic process in oocytes, we constructed the site-specific mutants targeting Serine 163 (S163) of FKBP25 (Dilworth et al., 2018). Ser163 was mutated to an alanine residue (S163A), to preclude phosphorylation, or to an aspartate residue (S163D), to mimic permanent phosphorylation (Hou et al., 2015). We injected the mRNA encoding FKBP25 mutants into GV oocytes for analysis. As shown in Figure 7A, immunoblotting verified that FKBP25-S163 mutant was efficiently expressed in oocytes. Compared with control groups, S163A mutant led to an almost 3-fold increase in meiotic abnormalities, such as spindle defects and chromosome misalignment (Figure 7B). Subsequently, we checked whether S163D mutant could rescue some phenotypic abnormalities of aged mouse oocytes. We injected the S163D mutant into old oocytes, and then they were *in vitro* matured for analysis (Figure 7C). It is worth noting that those meiotic phenotypes of old oocytes could be partially prevented by overexpression of phospho-mimetic S163D (Figures 7D,E). The results suggest that FKBP25 phosphorylation is essential for mediating the effects of aging on oocyte quality.

## Discussion

FK506 binding proteins 25 immunophilin, a crucial MAP, promotes MT polymerization and maintains chromosome stability (Wochnik et al., 2005). FKBP25 is localized to the nucleus, binds to DNA and interacts with chromatin modifying enzymes (Prakash et al., 2015, 2016). In addition, FKBP25 was detected on purified spindles in ovary cells (Bonner et al., 2011). In this study, a dynamic localization of FKBP25 was observed during mouse oocyte maturation. The colocalization of FKBP25 with α-tubulin further suggested that FKBP25 may participate in spindle organization during meiosis (Figure 1). Silencing of FKBP25 in U2OS cells displayed cell cycle delays, showing MT defects (Dilworth et al., 2018). Similarly, our results showed that FKBP25 depletion leads to some critical meiotic defects, including reduced polar body extrusion, aberrant spindle morphology and chromosome misalignment (Figures 2, 3). The SAC makes oocytes arrest at meiosis I stage when they encounter unattached chromosomes and abnormal spindle tension (Vogt et al., 2008; Polanski, 2013). Our study uncovered that K-MT connection is impaired, and SAC is activated in FKBP25-KD oocytes (Figure 4). In accordance with this observation, the frequency of aneuploidy was significantly increased in FKBP25-KD oocytes relative to controls (Figure 3). All these findings demonstrated that FKBP25 is crucially involved in the regulation of the assembly of meiotic structure during oocyte maturation. The decline of oocyte competence caused by maternal aging is the main factor in human infertility (Hamatani et al., 2004). It has been well documented that MT dynamics are altered in old oocytes (Luke et al., 2012; Nakagawa and FitzHarris, 2017). In this study, FKBP25 protein level was found to be decreased in oocytes from aged mice (Figure 5). Moreover, FKBP25 overexpression to some extent improved spindle assembly and increased euploidy in eggs (Figure 6). These findings above may provide a potential and effective way to obtain more high-quality oocytes for assisted reproductive technology (ART) or indirectly improve the fertility of aged females.

FK506 binding proteins 25 has a picturesque competence to bind nucleic acids and MTs, with the former regulated by PKC phosphorylation [17, 45, 46]. It has been documented that PKC could phosphorylate many of residues of FKBP25 during cell cycle. In particular, phosphorylation of FKBP25 disrupts its DNA binding ability (Dilworth et al., 2018). Here, we found that overexpression of S163A mutant could result in similar phenotypes to those observed in old oocytes. Of note, phosphorylation-mimetic mutant S163D markedly decreased the spindle/chromosome defects in aged oocytes (Figure 7). Collectively, FKBP25 phosphorylation might be a critical modification regulating the effects of maternal aging on oocyte quality.

In sum, several pieces of evidence in this study reveal that FKBP25, as a MAP, plays a pivotal role in the assembly of meiotic apparatus and maturational progression during oocyte meiosis. The results of this study provide a theoretical possibility for the application of FKBP25 to improve the fertility and ART efficiency of aged women.

## Materials and Methods

All chemicals and culture media were purchased from Sigma (St. Louis, MO, United States) unless stated otherwise. Each experiment was repeated at least three times.

### Mice

Three-4-week female mice were used as a control in this study. 42–45-week-old mice which near the end of their reproductive lifespan were used as a reproductive aging model. All animal work and experiments were carried out according to relevant ethical guidelines and regulations, and approved by the Animal Care and Use Committee of Nanjing Medical University.

### Antibodies

Rabbit polyclonal anti-FKBP25 antibody were purchased from Abcam (Cat#: ab16654; 1:150); Mouse monoclonal FITC conjugated anti α-tubulin antibody were purchased from Sigma (Cat#: F2168; 1:500); human anti-centromere CREST antibody was purchased from Antibodies Incorporated (Cat#: 15234; 1:500); Cy5-conjugated donkey anti-human IgG and FITC-conjugated donkey anti-goat IgG were purchased from Jackson Immuno-Research Laboratory (Cat#: 709605149 and 705095147; 1:500); Goat polyclonal anti-BubR1 antibody and mouse monoclonal anti-Myc tag antibody were purchased from Abcam (Cat#: ab28193 and ab18185; 1:250); and FITC conjugated goat anti-rabbit IgG purchased from Thermo Fisher Scientific (1:300).

### Collection and Culture of Oocytes

Oocytes were retrieved from female mice at the age of 3–4 week (young mice) and 42–45 week (reproductively old mice). To collect fully grown GV oocytes, female mice were superovulated with 5 IU pregnant mare serum gonadotropin (PMSG) by intraperitoneal injection. After 48 h, cumulus-oocyte complexes were acquired by manually rupturing of antral ovarian follicles. Cumulus cells were removed by repeatedly mouth pipetting. For *in vitro* maturation, GV oocytes were cultured in M16 medium under mineral oil at 37°C in a 5% CO2 incubator.

### Plasmid Construction and cRNA Synthesis

Total RNA was extracted from 100 mouse oocytes using Arcturus PicoPure RNA Isolation Kit (Applied Biosystems, CA, United States), and the cDNA was generated with QIA quick PCR Purification Kit (Qiagen, Germany). Plasmid construction and cRNA synthesis were conducted as we reported previously (Zeng et al., 2018). For the synthesis of cRNA, plasmids were linearized by NotI, and cRNAs were made using *in vitro* transcription with SP6 mMessage mMachine (Ambion, CA, United States) according to the manufacturer’s instruction and purified by RNeasy Micro Kit (Qiagen, Germany). Synthesized RNA was aliquoted and stored at −80°C. The related primer sequences can be found in Supporting Information Supplementary Table 1.

### Knockdown and Overexpression Experiments

Microinjection experiments were conducted using a Narishige microinjector. Microinjection of siRNA or cRNA was used to knock down or overexpress FKBP25 in mouse oocytes, respectively. 2.5 pl FKBP25 siRNA (1 mM) was injected into oocytes for knockdown analysis, or an equivalent amount of negative siRNA. 10 pl cRNA (10 ng/μl) was microinjected into GV oocytes for overexpression experiments. Following multiple washes, oocytes were arrested at GV stage in M16 medium containing 2.5 μM milrinone for 20 h to promote mRNA degradation or translation, and then cultured in M16 medium without milrinone for further experiments. The related primer sequences can be found in Supporting Information Supplementary Table 1.

### Western Blotting

A total of 100 GV oocytes were lysed in 2× Laemmli sample buffer containing protease inhibitor, and denatured at 95°C for 5 min, then frozen at −20°C until use. Total oocyte proteins were separated by 10% SDS-PAGE and electrophoretically transferred to PVDF membrane. Membranes were blocked with 10% nonfat milk in Tris-buffered saline containing 0.1% Tween-20 (TBST) for 1 h at room temperature and then probed with primary antibodies (Myc antibody, 1:1,000; FKBP25 antibody, 1:1,000) at 4°C overnight. After washes in TBST for three times, the membranes were incubated with HRP-conjugated secondary antibodies. Then, the protein bands were visualized using an ECL Plus Western Blotting Detection System (GE Healthcare, Little Chalfont, United Kingdom). Tubulin was used as a loading control.

### Immunofluorescence

Oocytes were fixed with 4% paraformaldehyde in PBS (pH 7.4) for 20 min at room temperature, permeabilized in 0.5% Triton-X 100 for 15 min at RT. Then, oocytes were blocked with 1% BSA-supplemented PBS for 1 h at RT and were subjected to indirect immunofluorescence staining by incubating with primary antibodies overnight at 4°C as follows: anti-FKBP25 antibody. To visualize spindle, oocytes were probed with FITC-conjugated tubulin antibody. Oocytes were co-labeled with CREST (1:500) to detect kinetochores. Following three washes, oocytes were labeled with secondary FITC- or Cy5-conjugated anti-body for 1 h at room temperature. Chromosomes were evaluated by staining with PI or Hoechst 33342 for 10 min. After washed in PBS, oocytes were mounted on antifade medium (Vectashield, Burlingame, CA, United States) and examined under a laser scanning confocal microscope (LSM 700, Zeiss, Germany).

### Chromosome Spread

Chromosome spread was conducted as described previously (Gao et al., 2018). To remove zona pellucida, MII oocytes were placed in Tyrode’s buffer (pH 2.5) for about 30 s at 37°C. After recovery in M16 medium for 10 min, oocytes were fixed in a drop of 1% paraformaldehyde with 0.15% Triton X-100 on a glass slide. After air drying, oocytes were incubated with CREST overnight at 4°C and then Cy5-conjugated secondary antibody for 1 h for kinetochore labeling. Chromosomes were stained with Hoechst 33342. Samples were examined under a laser scanning confocal microscope.

### Statistical Analysis

All experiments were replicated at least three times, and the data obtained were subjected to statistical analysis. Data are presented as mean ± SEM, unless otherwise indicated. Differences between two groups were analyzed by Student’s *t* test. Multiple comparisons between more than two groups were analyzed by one-way ANOVA test using GraphPad Prism 8. *P* < 0.05 was considered to be significant.

## Data Availability Statement

The original contributions presented in the study are included in the article/Supplementary Material; further inquiries can be directed to the corresponding author/s.

## Ethics Statement

The animal study was reviewed and approved by Animal Care and Use Committee of Nanjing Medical University.

## Author Contributions

QW designed and conceived the experiments. DW, HS, JZ, ZH, CL, LH, YX, ST, and JG performed the research and analyzed the data. QW and DW interpreted the data and wrote the manuscript. All authors have reviewed manuscript.

## Conflict of Interest

The authors declare that the research was conducted in the absence of any commercial or financial relationships that could be construed as a potential conflict of interest.
