# Supplementary material for: FKBP25 Regulates Meiotic Apparatus During Mouse Oocyte Maturation
Source: Front Cell Dev Biol. 2021 Jan 21;9:625805. doi: 10.3389/fcell.2021.625805 (PMC7859338; doi:10.3389/fcell.2021.625805)
Supplement: Supplementary file 1 [file Table_1.DOC]

# Supplemental Table 1

# Primer sequences of genes for cDNA amplification

| *Gene* | *Primer sequence* |
| --- | --- |
| FKBP25  (1st round) | Forward primer: 5’-GCAGCAGCAGGGGAAGATG-3’  Reverse primer: 5’- AAGGAAAGCAGCTCTCTGGAATACT-3’ |
| FKBP25  (2nd round) | Forward primer: 5’- GGGGGCCGGCCGCAGCAGCAGGGGAAGATG-3’  Reverse primer: 5’- GGGGGCGCGCCTCAGTCAATGTCTACTAATTCCACT-3’ |
| FKBP25-S163A | Forward Primer: 5’-TAGCTTTTAAGGTCGGAGTAGGCAAGGTTATCA- 3’  Reverse Primer: 5’-CTCCGACCTTAAAAGCTAAAGGTTTGGCATTTTTCTTCTTC- 3’ |
| FKBP25-S163D | Forward Primer: 5’-TAGATTTTAAGGTCGGAGTAGGCAAGGTTATCA - 3’  Reverse Primer: 5’-CTCCGACCTTAAAATCTAAAGGTTTGGCATTTTTCTTCTTC-3’ |

# FKBP25 siRNA and control siRNA sequences

| *Gene* | *siRNA sequence* |
| --- | --- |
| FKBP25 | Forward: 5’-GCUGGUAUACAGGGACACUTT- 3’  Reverse: 5’-AGUGUCCCUGUAUACCAGCTT-3’ |
| Control | Forward: 5’-UUCUCCGAACGUGUCACGUTT-3’  Reverse: 5’-ACGUGACACGUUCGGAGAATT-3’ |
